# Improving Human–Robot Interaction by Enhancing NAO Robot Awareness of Human Facial Expression

**DOI:** 10.3390/s21196438

**Published:** 2021-09-27

**Authors:** Chiara Filippini, David Perpetuini, Daniela Cardone, Arcangelo Merla

**Affiliations:** Department of Neurosciences, Imaging and Clinical Sciences, University G. d’Annunzio of Chieti-Pescara, 9, 66100 Chieti, Italy; chiara.filippini@unich.it (C.F.); david.perpetuini@unich.it (D.P.); d.cardone@unich.it (D.C.)

**Keywords:** facial expression recognition, emotion recognition, human–robot interaction, affective computing, machine learning

## Abstract

An intriguing challenge in the human–robot interaction field is the prospect of endowing robots with emotional intelligence to make the interaction more genuine, intuitive, and natural. A crucial aspect in achieving this goal is the robot’s capability to infer and interpret human emotions. Thanks to its design and open programming platform, the NAO humanoid robot is one of the most widely used agents for human interaction. As with person-to-person communication, facial expressions are the privileged channel for recognizing the interlocutor’s emotional expressions. Although NAO is equipped with a facial expression recognition module, specific use cases may require additional features and affective computing capabilities that are not currently available. This study proposes a highly accurate convolutional-neural-network-based facial expression recognition model that is able to further enhance the NAO robot’ awareness of human facial expressions and provide the robot with an interlocutor’s arousal level detection capability. Indeed, the model tested during human–robot interactions was 91% and 90% accurate in recognizing happy and sad facial expressions, respectively; 75% accurate in recognizing surprised and scared expressions; and less accurate in recognizing neutral and angry expressions. Finally, the model was successfully integrated into the NAO SDK, thus allowing for high-performing facial expression classification with an inference time of 0.34 ± 0.04 s.

## 1. Introduction

Artificially intelligent agents such as social robots (SRs) have increased in popularity over the last few decades and are expected to be used in a variety of social applications. SRs can currently execute a variety of tasks autonomously, which has resulted in their continuous introduction into society. SR applications that are becoming available include therapy for autistic children [1], exercise coaches [2], specialized education [3], and assistance to elderly persons affected by dementia [4]. Moreover, studies have shown that a robot can affect its social environment beyond the person who is interacting with it [5,6]. For example, studies of robots used in autism therapy have demonstrated that robots can influence the way children interact with others [1], just as many previous works have shown that employing robots in the rehabilitation field has considerable effects on patient improvement [7]. In all these applications, robots are usually designed to interact with humans in a spontaneous, interpersonal way, often with specific social–emotional goals [8]. However, these agents are still struggling to interact with users in a human-like way, which is an issue that has proved to be challenging to overcome. Just as human–human interaction can be discouraged by the absence of initiative from one of the parties, human–robot interaction can fall short if there is limited or no commitment by the robot or the human user. At the same time, to take initiative, the robot must be able to choose what actions to perform and to what extent. Additionally, it should adapt its behaviors based on the user’s reactions and possibly produce empathetic responses to the emotions of the person it is interacting with. Indeed, empathy is a crucial component of human–human communication, so replicating it for SRs is important. As a result, the first step towards achieving human-like interaction skills is for the robot to adequately recognize the user’s emotional state and accordingly adjust its responses, thus mimicking human empathetic behaviors [9].

As with person-to-person communication, facial expressions are the privileged channel for recognizing an interlocutor’s emotional expressions [10]. The analysis of the expressive component of emotion starts from the assumption that different emotions are correlated with specific configurations of the face. Different models or theories have been developed and used by psychologists or cognitive neuroscientists to distinguish between emotions based on facial expressions. The categorization of human emotions is mainly based on two perspectives: discrete and dimensional [11]. In the dimensional perspective, emotions are represented by the valence and arousal dimensions [12]. The valence dimension indicates the intrinsic attractiveness or aversion of an event, object, or situation, and it varies from negative to positive [11]. Arousal, on the other hand, indicates whether the subject is responsive at that given moment and for that given stimulus, as well as how active he/she is. Conversely, from the discrete perspective, all humans are thought to have an innate set of basic emotions that are cross-culturally recognizable. Indeed, several researchers have proposed that people display universal prototypical facial expressions that are specific to basic emotions [13]. Although facial expression recognition (FER) has been widely studied and reached significant progress, recognizing such expressions under naturalistic conditions is more challenging. This is mainly due to variations in head pose and lighting conditions, as well as occlusion, and because unposed expressions are frequently subtle. Convolutional neural networks (CNNs) have the potential to overcome these challenges. Indeed, the advent of CNNs as appearance-based classifiers has substantially benefited several well-known challenges in computer vision in recent years. On various acknowledged benchmarks, tasks like object identification [14], face recognition [15], and object detection [16] have experienced significant improvements. Additionally, different recent works on FER have successfully utilized CNNs for feature extraction and inference (e.g., [17,18,19]). This article proposes a CNN designed to enhance social robots’ capability to recognize facial expressions related to emotions in real-time and realistic conditions. The developed CNN-based FER model can be successfully embedded into a humanoid robot platform. In addition, through the analysis of the developed CNN outcomes, information about the arousal dimension of an interlocutor’s emotions can be provided to the robot. This research was focused on the humanoid NAO robot platform because it is a social robot (https://www.softbankrobotics.com/emea/it/nao accessed on 25 September 2021).

The NAO robot is one of the most widely used agents for human interaction thanks to its design and open programming platform. In fact, NAO’s pleasant voice and friendly aspect aids in providing a better user experience. NAO is currently being employed in applications including rehabilitation sessions, elderly healthcare, and interactions with young people [20,21], where understanding human emotional expression is of paramount importance to foster human interaction. Although the NAO robot is equipped with an FER module, certain experimental conditions or use cases may require additional functionality and affective computing capabilities not already available in the module. Therefore, it is crucially important to determine the NAO’s FER module accuracy, as well as to expand and further enhance the NAO robot’s awareness of human facial expression. To achieve this task, the CNN implemented in this study was trained and tested with a validated FER dataset. We used the FER2013 dataset, which is the most common image dataset in CNN-based FER and one of the largest publicly available datasets in this field [22]. However, the main reason for selecting this dataset was its relevance to realistic conditions. 

**Figure 1:**
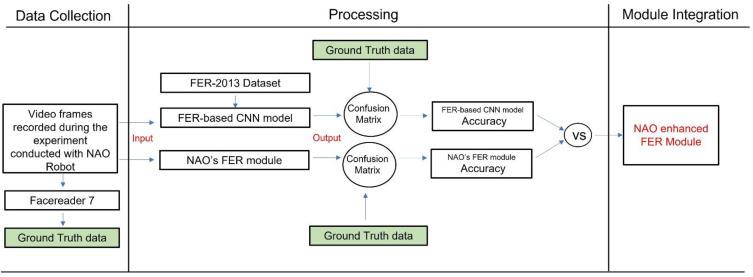
The overall paper processing workflow.

This dataset was employed to train and test the accuracy of the developed CNN-based FER model. Furthermore, we conducted an emotion recognition experiment with the NAO robot in a real-world context to test the NAO’s capacity to recognize facial expressions as well as the NAO’s FER module’s accuracy. The data collected during the experiment were also analyzed with the developed CNN-based FER model. To compute the accuracy of both models (the CNN-based FER module and the NAO’s FER module), the data recorded during the experimental sessions were also analyzed with the FER software, i.e., FaceReader 7 (developed by VicarVision and Noldus Information Technology, https://www.noldus.com/human-behavior-research/products/facereader accessed on 25 September 2021) already used in the literature [8] and validated in various research studies [23]. Such software provided the ground truth data on FER and arousal detection. The CNN-based FER module outcome was also used to determine the arousal level of the interlocutor’s emotional expression. Finally, the developed model was integrated into the NAO platform under its Windows Python SDK and allowed to enhance the NAO robot’s awareness of human facial expression. The presented paper’s processing workflow is shown in Figure 1. 

In conclusion, the novelties that this study aimed to introduce are the integration of a CNN-based FER model into the NAO robotic platform and its evaluation in real-world human–robot interaction tasks. Since social robots are still struggling to interact with users in a human-like way, which is a challenge that has proven difficult to overcome, it is essential to test the robotic system’s recognition of human facial expression in real-life situations. Additionally, the CNN presented in this study strives to endow, for the first time, the NAO robot with the capability to estimate the interlocutor’s arousal level in real time, which is of paramount importance to ensure better interactions between the robot and humans.

## 2. Materials and Methods

### 2.1. Participants

Twenty-four adults (ages: 27–38; mean: 30.4) participated in the experiment. Among them, nineteen adults were recruited to test the CNN-based FER model and NAO’s FER module on human adults and five were recruited to validate the CNN-based FER model integrated into the NAO robot. Participation was strictly voluntary. Participants were adequately informed about the study’s purpose and protocol prior to the start of the experimental trials, and they signed an informed consent form outlining the methods and purposes of the experimentation in accordance with the Declaration of Helsinki.

### 2.2. Experimental Session

**Figure 2:**
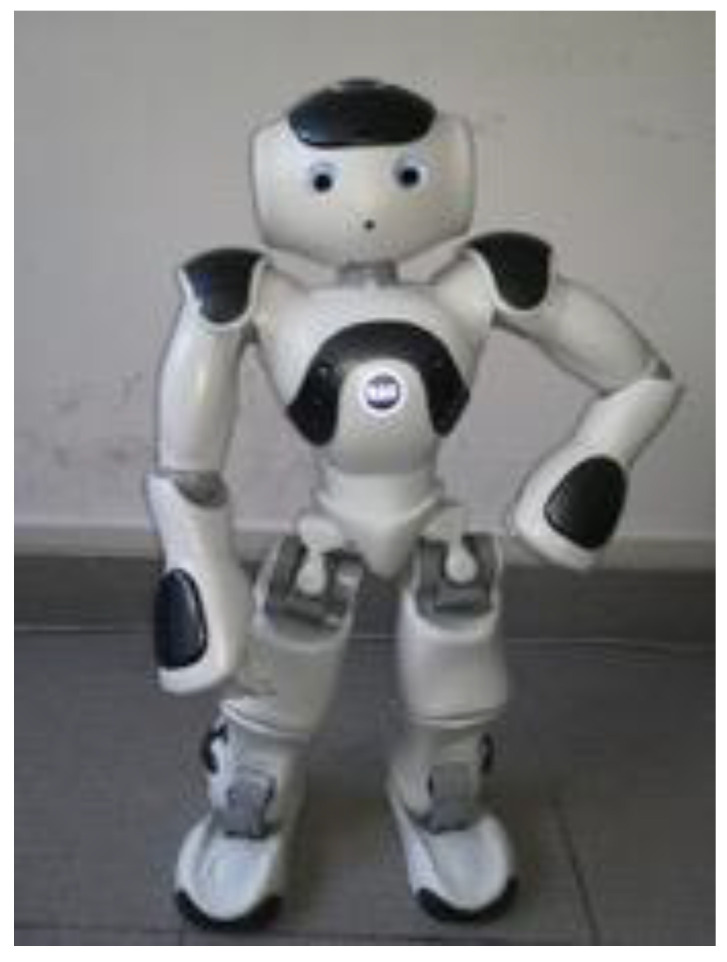
NAO robot v6 from SoftBank Robotics employed in the experiment.

The experimental session was conducted using the humanoid robot NAO from SoftBank Robotics. NAO is 58 cm high and has a number of sensors and abilities such as those of moving, seeing, speaking, hearing, and limited manipulation of objects (Figure 2). The version of the robot used in this research was the latest NAO, v6. NAO has Python SDK (Naoqi) available to allow researchers to develop advanced intelligent components for robot motion processing, speech, and vision [24]. The robot is equipped with two built-in cameras, one located at the mouth level and the other located on its forehead. Both are 920p cameras able to run at 30 images/second for (up to) 1280 × 720 pixel images. NAO’s head can move 68 degrees vertically and 239 degrees horizontally, and its camera can see 61° horizontally and 47° vertically. Hence, it has good vision of its environment. Its forehead camera was used to record videos of the person in front of NAO during the experimental session. The robot platform also provides vision APIs (application programming interfaces) for image processing, movement detection, background darkness checking, and estimating the emotions of the human in front of the robot (described in Section 2.3). 

**Figure 3:**
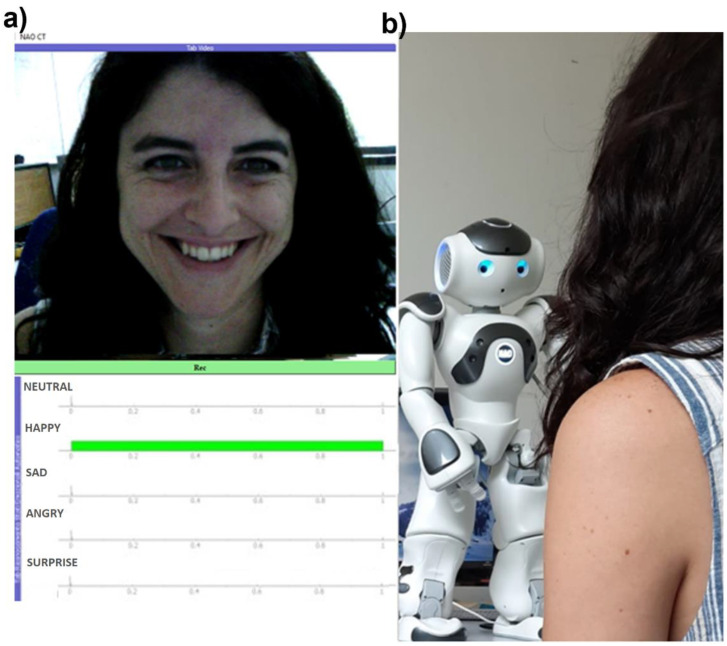
(**a**) Representation of the developed GUI reporting the image acquired from the NAO’s forehead webcam and NAO’s FER module outcome in real-time. (**b**) Interaction between NAO robot and a representative participant.

The experimental protocol was structured in two phases. The first phase was familiarization, where NAO introduced itself and the purpose of the experiment, as well as asking the participants for their willingness to participate. The familiarization period lasted around 2 min. The second phase consisted of NAO asking the participants to express and maintain facial expressions related to five basic emotions (i.e., happy, sad, anger, fear, and surprise), as well as a neutral expression, for 20 s. Moreover, it instructed the participants to show their facial expressions in a natural way. This second phase lasted around 3 min, so the total duration of each experimental session was 5 min. The participant sat in front of the robot (see Figure 3b) and followed its instructions without any help from the interlocutor. NAO was programmed to perform the described protocol through its SDK (Naoqi 2.8.6 Python version). The facial expressions analyzed in this study are related to Ekman’s classification of basic emotions [25]. 

In addition, five participants were recruited to validate the real-time application of the CNN-based FER model integrated into the NAO robot in terms of accuracy achieved during the human–robot interaction and the time required to process the emotional outcome. Participants were invited to familiarize themselves with the robot and then perform neutral and five basic facial expressions (i.e., happy, sad, angry, scared, and surprised), as requested by NAO. Furthermore, considering that the target person’s frontal views are not always properly captured in many real-life robotic interaction situations, an appropriate CNN-based FER system integrated into a robotic platform should be able to distinguish emotions from different facial angles. Therefore, to evaluate how the integrated model functioned when the participants were naturally moving, NAO asked participants to maintain the required facial expressions while slowly rotating their heads left and right and up and down. 

### 2.3. NAO’s FER Module

The NAO robot has a dedicated API (ALMood) that provides information on the instantaneous emotional state of the speaker. The API emotional processing is built upon various extractors. In particular, it uses a vision module called ALFaceDetection to detect and optionally recognize faces in front of it, a module called ALGazeAnalysis to evaluate the head angles, and a module called ALFaceCharacteristics to analyze the face and detect facial expressions. ALMood retrieves information from the abovementioned extractors to combine them into high-level indicators classified within the following facial expressions: happy, sad, surprised, angry, and calm/neutral. API extractors can be triggered with a processing frequency set by the operator. In this study, the processing frequency was set to 1 Hz.

A graphical user interface (GUI) named “NAO Control Terminal (CT)” was developed in this study to visually check the accuracy of the NAO’s FER module and to allow the experimenter to have real-time awareness of NAO’s vision and processing results. The implemented GUI is shown in Figure 3a. The real-time acquisition of the images from the NAO’s forehead webcam was performed by subscribing to the NAO ALVideoDevice API. From NAO CT GUI, it was also possible to start and stop the experimental session recording. 

### 2.4. FER2013 Dataset

The dataset used to train and test the CNN-based FER model was the FER2013 dataset. Even though there are several video datasets with much higher numbers of frames, the number of subjects in such datasets is small and the frames are naturally more highly correlated. However, the main reason for choosing this dataset was its relevance to realistic conditions. Indeed, the dataset is very challenging to analyze because the depicted faces significantly vary in terms of pose, face, age, expression intensity, and other factors. FER2013 was created by Pierre Luc Carrier and Aaron Courville, and it consists of 35,887 face crops [26]. All samples are labeled with basic expressions. The images are grayscale and have a 48 × 48 pixels resolution. Human accuracy for this dataset was found to be around 65.5% [26].

### 2.5. CNN-Based FER Model

**Figure 4:**
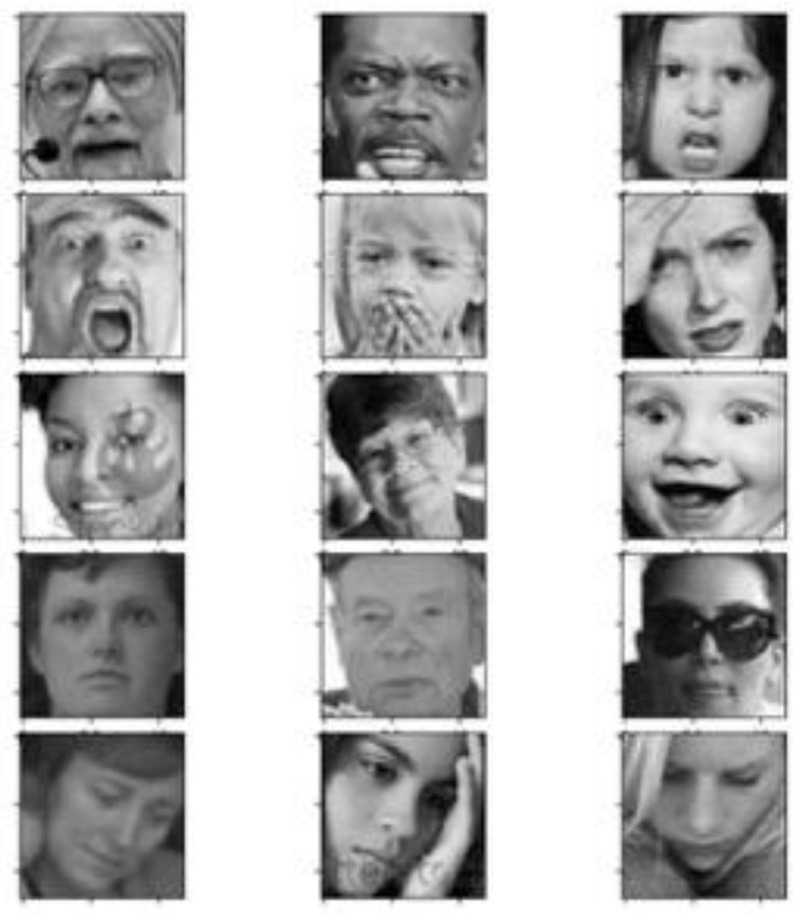
FER2013 [26] dataset sample images. Such images highlight variations in illumination, pose, expression intensity, age, and occlusions typical of the dataset.

The CNN-based FER model was trained and tested on the FER2013 dataset (Figure 4). The images employed in this analysis were related to the following expressions: angry, scared, happy, neutral, sad, and surprised. Before applying the CNN algorithm, a pre-processing step was conducted on the input images; to ensure facial geometric correspondence and keep face size constant across the dataset images, affine transformation (rotation, scaling, and translation) was performed on the input images. Affine transformation is usually used to normalize face geometric position and maintain face magnification invariance [27]. The preprocessed images were then used to feed the CNN. The CNN structure employed in this work was heuristically chosen in a similar fashion to previously reported CNN structures in FER analysis [22].

**Figure 5:**
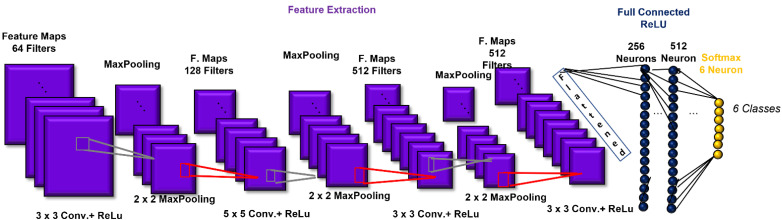
Convolutional-neural-network (CNN) architecture, which includes the structure of the implemented convolution building block.

The CNN architecture consisted of 4 convolutional layers, 4 pooling layers, and two layers fully connected prior to the output layer. The first convolutional layer was composed of 64 filters (size: 3 × 3) applied to the input images to generate 64 feature maps of the images. The second, third, and fourth convolutional layers were composed of 128 (size: 5 × 5), 512 (size: 3 × 3), and 512 (size: 3 × 3) filters, respectively. After each convolutional layer, batch normalization was introduced. The activation function employed in all 4 convolutional layers and both fully connected layers was the rectified linear unit (ReLU) function, which we used to add non-linearity to the network [28]. Then, as a down-sampling or pooling layer, MaxPooling was selected, with the largest element from the rectified feature map retained. A filter size of 2 × 2 was implemented for all the MaxPooling layers. The two fully connected layers consisted of 256 and 512 neurons. The fully connected layers were employed to summarize information and compute the class score. Lastly, a SoftMax function was used in the output layer to output a probability value from 0 to 1 for each of the six classification classes (angry, scared, happy, neutral, sad, and surprised). All the biases of the CNN were initialized to a small constant, i.e., 0.1, whereas the weights were initialized in a pseudo-random manner by employing a truncated normal distribution (standard deviation = 0.1). Finally, the implemented loss function was categorical cross-entropy. The CNN architecture is shown in Figure 5. 

The developed architecture was then boosted through an optimization procedure. The model optimization was primarily focused on reducing overfitting because overfitting is one of the main risks that can be incurred when training a CNN. A technique used to address overfitting is regularization [29]. In this study, the dropout regularization method was performed; during training, units (along with their connections) were randomly removed from the neural network. This prevented units from excessively co-adapting. Moreover, to reduce the internal covariate shifts and instability in distributions of layer activation functions, batch normalization was used [30]. This was intended to reduce model complexity and make the model less prone to overfitting. To address the model generalization performance, a ten-fold cross-validation procedure was performed. Finally, instead of using a fixed learning rate hyperparameter, which could lead the model to converge too quickly to a suboptimal solution, a tunable learning rate was implemented over the training process. In detail, a function was implemented to reduce the learning rate by a factor of 0.1 once learning stopped improving after at least 10 epochs. 

The optimization procedure was iterated for 90 epochs, with a batch size of 128 samples. The metric used for evaluating the model was accuracy. Accuracy represents the percentage of correct predictions out of the total number of test samples. Such metrics were evaluated by counting the number of correct CNN predictors after an argmax evaluation of the CNN output vector and averaging them among the plateau iterations. The described CNN model was implemented in Python using the Keras API with the TensorFlow backend. For model evaluation, the scikit learn library was utilized. 

**Figure 6:**
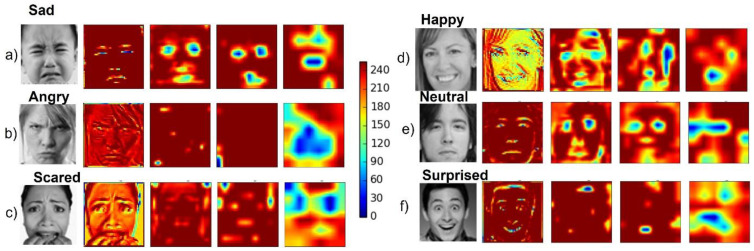
Convolutional neural network (CNN) heatmaps. Representative input image of the FER2013 dataset along with the related heatmap resulting from each of the four convolutional layers for images labeled (**a**) sad, (**b**) angry, (**c**) scared, (**d**) happy, (**e**) neutral, and (**f**) surprised.

Finally, for each CNN convolutional layer, an average heatmap was implemented to visualize the salient image regions used by the CNN to reach its final classification decision (Figure 6). 

### 2.6. NAO’s FER and CNN-Based FER Models Accuracy Assessment on Experimental Data

The NAO’s FER module outcome was saved as a json file for each participant. The experimental data recorded through the NAO CT GUI were also analyzed with the FaceReader 7 software and the CNN-based FER model. FaceReader 7 is a commercial software developed to automatically classify static (i.e., still pictures) and dynamic (i.e., videos) facial expressions. According to research validation studies, FaceReader 7 shows the best performance out of the major currently available software tools for emotion classification [31,32]. The software has been demonstrated to be robust under varying conditions of pose, orientation, and lighting using an implementation of the Active Appearance Model as core technology [31]. FaceReader 7 classifies people’s emotions into the discrete categories of basic emotions, but it can also analyze the arousal dimension of an emotion based on the circumplex model of affect [33]. Basic expressions such as happy, neutral, angry, scared, sad, and surprised, as well as arousal level, were extracted from each frame of the video based on the software analysis results. Such results were then down-sampled to 1 Hz to be compared with the NAO’s FER module outcome. Moreover, since the NAO’s FER module did not consider the facial expression related to fear, the frames that the FaceReader software classified as scared were not included in this comparison.

The CNN-based FER model developed in this study was also tested on the same experimental videos. In detail, such model was previously saved using Keras python library, which provides the ability to describe any model using the json format with a *to_json()* function. This can be saved to a file and later loaded via the *model_from_json()* function that creates a new model from the json specification. The weights are directly saved from the model using the *save_weights()* function and later loaded using the symmetrical *load_weights()* function. However, to successfully apply the model to new images, the images need to be equivalent in size (48 × 48) and content to the training images. Therefore, the video frames were firstly analyzed and processed with OpenCV, an open-source computer vision library [34]. The processing pipeline implemented to extract data from recorded video images suitable for feeding the developed CNN-based FER model and obtaining the model outcomes was as follows:

- Reading each video frame.
- Converting the color image to grayscale, since the model only works on grayscale images.
- Detecting the participant face within the image by using OpenCV pre-trained face detection classifier.
- Extracting the rectangular region where the face was found and resizing such region to 48 × 48 pixels.
- Applying the loaded CNN-based FER model to the image.
- Collecting model outcome.

The model outcome was also down-sampled to 1 Hz.

To assess the accuracy of the CNN-based FER model and the NAO’s FER module, as well as to evaluate the effectiveness of both approaches, a confusion matrix was computed to compare both approaches’ outcomes with the expected values (i.e., FaceReader 7 results). A confusion matrix is a performance metric commonly used in machine learning classification. Such a matrix compares the expected values with those predicted by the model under analysis. Using this provided a holistic view of how well the classification model was performing and what kinds of errors it was making. The confusion matrices of both NAO’s FER and CNN-based FER models are reported in Section 3.2.

### 2.7. Arousal Computing

The outcome of the CNN-based FER model applied to the experimental data was also used to predict the arousal level of the participants’ emotional responses. Specifically, the activation function of the CNN-based FER model output layer, i.e., the SoftMax function, assigned decimal probabilities to each output class (happy, neutral, angry, scared, sad, and surprised) for each input image. Such probabilities were used as regressors to predict the arousal level of a given image. A machine learning algorithm was utilized for the prediction task. In detail, a general linear model (GLM) was trained to predict the level of arousal obtained from FaceReader 7, which relies on such probabilities, through a supervised learning procedure. Although several machine learning approaches could be suited for such a purpose, the GLM was chosen to decrease procedural complexity due to the small number of available independent features and the exploratory nature of the implemented approach [35].

Because of the multivariate (6 regressors) GLM approach, the in-sample performance of the procedure did not reliably estimate the out-of-sample performance [36]. The generalization capabilities of the procedure were thus assessed through cross-validation. Specifically, a leave-one-subject-out cross-validation was performed. This cross-validation procedure consisted of leaving one subject (all the samples from the same subject) out of the regression and estimating the predicted output value on the given subject by using the other participants as the training set of the GLM model. The procedure was iterated for all the subjects. Correlation analyses were performed on the cross-validated GLM outcome and the arousal level obtained through the FaceReader 7 software.

### 2.8. Integration of CNN-Based FER Model with NAO Robot SDK

**Figure 7:**
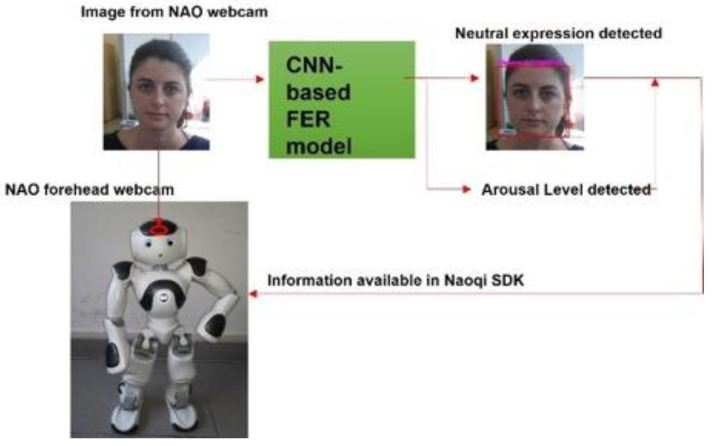
Information-processing workflow. The images were retrieved from NAO’s forehead webcam and analyzed through the CNN-based FER model. The model outcome, integrated into Naoqi SDK, was available for the robot to use.

The developed CNN-based FER model was applied to the images streaming from the NAO’s forehead camera under its Windows Python SDK (Naoqi). To access the NAO’s camera and read the image sequences, the ALVideoDevice API and the *getImageRemote()* function were utilized. Then, the image from the API was converted from QImage into matrix data that could be analyzed through the OpenCV library. The OpenCV was employed to detect the participant’s face and extract the rectangular region where the face was found. This region was then analyzed with the CNN-based FER model; the model outcome, as well as the rectangle where the face was found, of the analyzed image were then shown. In addition, the arousal level predicted by the GLM model, which relied on the CNN-based FER model outcome, was added as useful information that the robot could retrieve when needed. Figure 7 shows the information-processing workflow. 

Moreover, the performance of the CNN-based FER model integrated into NAO was assessed in terms of:

- Accuracy achieved during human–robot interaction activities (described in Section 2.8).
- Percentage of frames in which the interlocutor’s face was correctly tracked.
- Time required to process the facial expression detection.

In detail, the accuracy was computed by comparing the integrated model outcome with the expected values provided by FaceReader 7. Such accuracy evaluations were also performed with respect to participants’ different facial orientation angles. The facial orientation angles were computed from the head pose estimated using OpenCV. 

## 3. Results

### 3.1. CNN-Based FER Model Results for FER2013 Dataset

**Figure 8:**
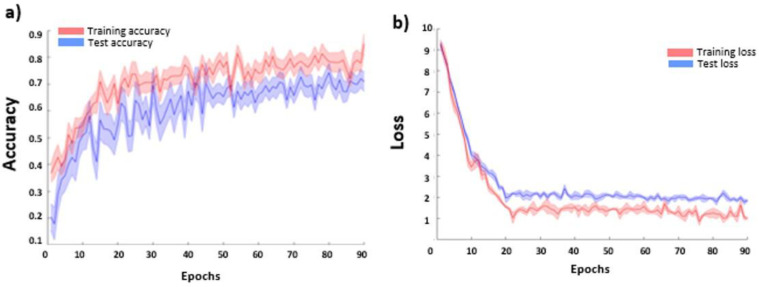
CNN average (and related standard errors) cross-validated (**a**) accuracy and (**b**) loss function values as a function of the training epoch for the training and test sets.

The CNN-based FER model average accuracies for the FER2013 dataset are reported in Figure 8a as a function of the training epoch for the training and testing sets. No over-fitting effect (decrease of the accuracy at increasing epoch) was visible in the testing set, proving the efficacy of the employed procedure. The average loss function values (and related standard errors) for the training and testing sets are also reported in Figure 8b. The CNN accuracy in the test sample reached a plateau value of 0.69 ± 0.03.

**Figure 9:**
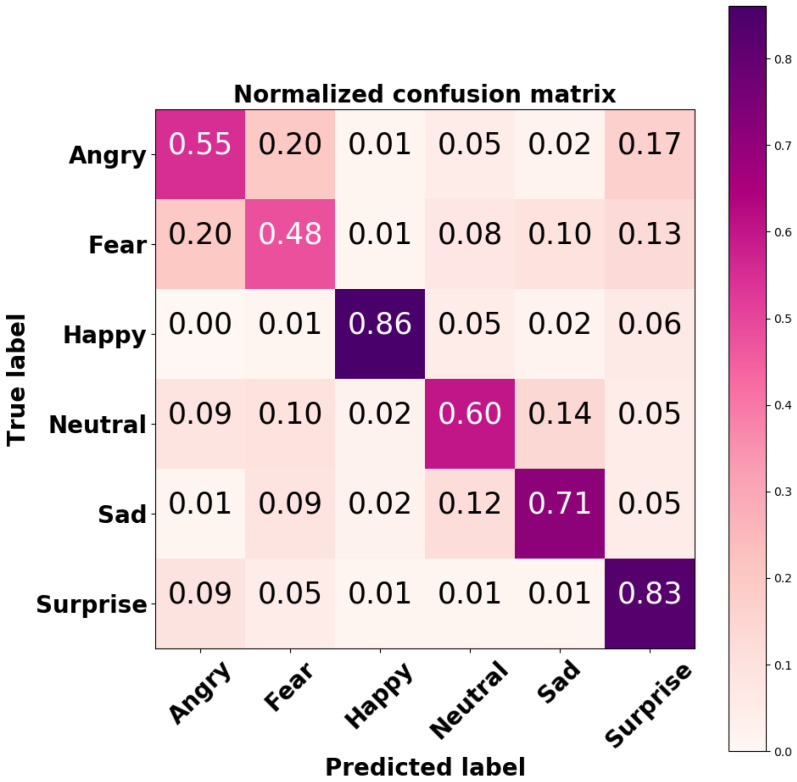
Normalized confusion matrix of the CNN-based FER model tested on the FER2013 dataset.

In order to explore the performance of the developed CNN on each output class, the confusion matrix was computed; it is shown in Figure 9. 

### 3.2. NAO’s FER and CNN-Based FER Models Results on Experimental Data

**Figure 10:**
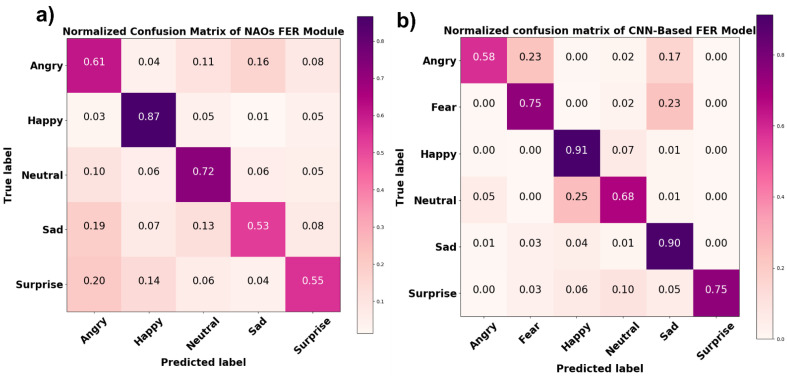
Normalized confusion matrix of (**a**) NAO’s FER module and (**b**) the CNN-based FER model tested on experimental data.

A comparison between the NAO’s FER module’s outcome on the experimental session data and the results of the FaceReader 7 software analysis of the same data showed an average accuracy of 65%. A normalized confusion matrix reporting the performance of each class is shown in Figure 10a. In addition to confusion matrix precision, recall and F1 score metrics were also computed for each class (Table 1). Precision represents the ratio of correctly predicted positive observations to the total predicted positive observations, recall is the ratio of correctly predicted positive observations to all true positive observations, and F1 score represents the weighted average of precision and recall. Figure 10b shows the normalized confusion matrix related to the comparison between the results of the CNN-based FER model and the FaceReader 7 software. The average accuracy achieved by the CNN-based FER model was 77%. The precision, recall, and F1 score of each class are reported in Table 1. 

### 3.3. Arousal Results

**Figure 11:**
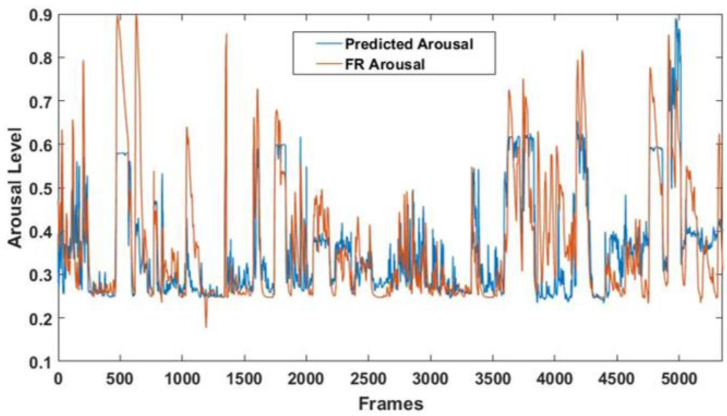
Arousal levels detected throughout the entire experimental session. The blue line represents the arousal predicted by the GLM model, and the red line represents the arousal detected by the FaceReader 7 software.

A significant correlation between the arousal detected by the FaceReader 7 software and the predicted arousal was obtained (r = 0.69; *p* = ~0), thus demonstrating the good performance of the multivariate analysis [37]. Figure 11 shows the arousal levels detected throughout the entire experimental session by the GLM model and the FaceReader 7 software. 

### 3.4. CNN-Based FER Model Integration into NAO Robot SDK

**Figure 12:**
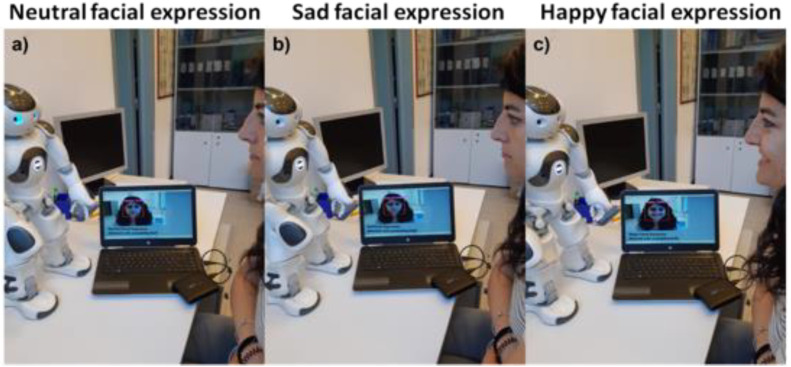
Pictures captured during the NAO’s interaction with a representative subject. The facial expressions detected by the NAO robot through the CNN-based FER model for (**a**) neutral, (**b**) sad, and (**c**) happy facial expressions are also displayed in the images.

The CNN-based FER model and the pipeline described in Section 2.8 were successfully applied to the image streaming of the NAO robot (source code available at https://dx.doi.org/10.6084/m9.figshare.16682476, accessed on 25 September 2021). Figure 12 depicts NAO’s interaction with a representative subject, demonstrating the model’s correct functioning and integration with the Naoqi SDK. In detail, Figure 12 shows three pictures captured during the interaction in which it is possible to observe that the images from the NAO’s forehead camera were successfully processed using the CNN-based FER model. Figure 12a–c shows the NAO robot (via the CNN-based FER model) detecting the neutral, sad, and happy facial expressions, respectively, of a subject. 

**Figure 13:**
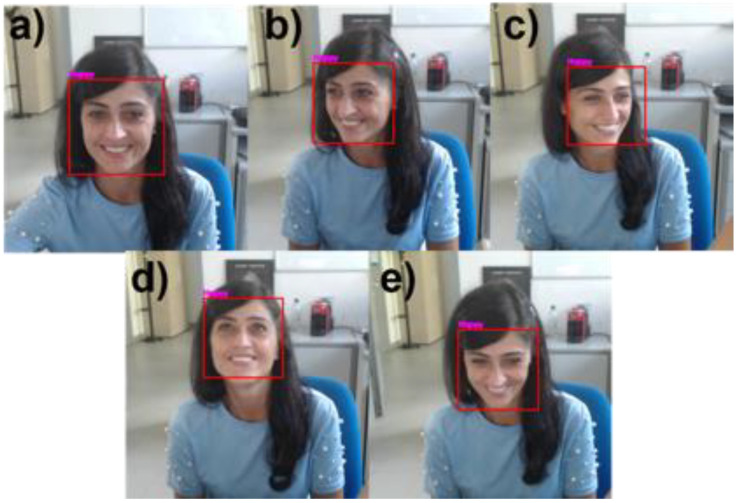
Pictures of a representative participant exhibiting happy facial expressions to the NAO robot in (**a**) frontal; (**b**,**c**) yaw; and (**d**,**e**) pitch orientations. The red rectangle represents the detected face, and the writing above the rectangle reflects the facial expression recognized by NAO through the integrated model.

Furthermore, Table 2 demonstrates the overall accuracy of the NAO robot’s integrated CNN-based FER model in detecting facial expressions during human–robot interaction tasks at different facial orientation angles. The table also includes the percentage of frames in which the face was correctly detected. Figure 13 depicts a representative participant performing a happy facial expression with different facial orientation angles. The red rectangle represents the detected face, which constitutes the input to the NAO-integrated CNN-Based FER model, and the facial expression recognized by the integrated model is reported above the rectangle. 

The facial expression inference time provided by the model and pipeline integrated into the NAO was 0.34 ± 0.04 s. By contrast, the ALMood NAO’s current API (described in Section 2.3) could take up to 2 s to load the API extractors and provide an expression recognition outcome.

## 4. Discussion

Over the last few decades, SRs have been widely employed in social applications for many purposes, such as education, health, and communication. However, some limitations have been observed during human–robot interaction, mainly due to non-natural cooperation between the two parties. To build robots that interact in a more intuitive and human-like fashion, the perception of human emotions is essential [38]. The development of such multimodal agent-based interfaces has greatly benefited from automatic face and expression recognition. However, detecting emotions from spontaneous facial expressions during real-life human–robot interaction may still be difficult due to pose and subject variations, lighting changes, occlusions, and background clutter. One of the most used SRs for human interaction purposes is the NAO robot, which has led human users to have high expectations for such a robotic platform concerning its social abilities. However, the original vision APIs provided by the robot’s SDK struggles to handle such challenging facial emotion recognition tasks [39]. This research was thus motivated to develop a highly accurate FER model for a humanoid robot to deal with emotions detection in real-life situations. Most importantly, the NAO’s FER module had yet to provide interesting affective computing functionalities. Therefore, the aim of the study was to enhance NAO’s FER module accuracy in detecting facial expressions and to provide the robot with information regarding the recognition of the fear emotion’s facial expression and the emotion’s arousal level

The CNN-based FER module developed for this purpose was trained and tested on the FER2013 dataset, a challenging dataset including images with variations in illumination, expression intensity, age, and occlusion. For this dataset, the implemented CNN model achieved accuracies of 86% and 83% for the happy and surprised facial expressions, respectively. These two expressions are also the most easily recognized by humans [40]. An accuracy of 71% was achieved for neutral facial expression, whilst lower accuracies were achieved for angry and scared facial expression; the model tended to mistake them for each other. However, this result is consistent with recent findings in the field [41,42]. The overall accuracy of our model on the FER2013 dataset was 69%, which is considered higher than the human-level accuracy of 65%. The heatmaps of the model’s convolutional layers (Figure 6) show that the model focused on important aspects of the input image, i.e., lips, eyes, eyebrows, and mouth. 

The accuracy of the CNN-based FER module considerably increased with the experimental data; it reached 91% and 90% for happy and sad facial expressions, respectively; 75% for surprised and scared expressions; and 65% and 58% for neutral and angry expressions, respectively. This validated the developed model’s generalization capability. However, it is worth noting that since during the experimental session, the participants were asked to maintain the same expression for 20 s, so the model’s input data in this task were more consistent and steadier than the pictures in the database, probably contributing to the improved model accuracy. On the other hand, this showed that increasing the number of more standardized input images in illumination and occlusions can help the model increase its accuracy, thus establishing a framework for future model improvement. NAO’s FER module was also tested with the same experimental data, and it demonstrated lower accuracy for the facial expressions it was able to detect compared to the developed CNN-based FER model. Specifically, the NAO’s FER module was able to detect happy, neutral, angry, surprised, and sad facial expressions with accuracies of 87%, 72%, 61%, 55%, and 53%, respectively. 

Finally, since the NAO’s FER module is unable to detect the arousal level of its human interlocutor, the CNN-based FER module was used to provide such information to the robot. Indeed, the output class probabilities resulting from the model were employed as input regressors for a GLM algorithm. The correlation between the arousal level detected by FaceReader 7 and the arousal predicted by the multivariate approach was r = 0.69 (*p* = ~0), indicating good arousal estimation through the considered regressors. Such information allows the robot to better characterize its interlocutors’ emotional state during real-time interactions. 

The overall system was integrated with the Python SDK of the latest NAO v6 robot. In detail, the NAO vision API was used to capture the images streaming from the NAO;s forehead webcam; such images were then analyzed through the developed CNN-based FER model, and the model outcomes were made available to the robot. The integrated model enhanced NAO’s FER module in facial expression analysis and provided information to the robot about the scared facial expression and the emotions’ arousal levels. Moreover, its inference time boosted the NAO performance in facial expression recognition from 0.5 frames per second (FPS) to 3 FPS, thus allowing the interaction to remain fluid in real-world scenarios. As a result, the developed model has the potential to lead NAO robot in approaching human-like interaction skills by accurately recognizing users’ emotional states and allowing it to use such information to accordingly adjust its responses. In future studies, we are interested in validating the enhanced NAO’s FER module with challenging populations such as infant, elderly, or sick people in order to make the NAO robot more responsive to their needs.

### Novelty and Limits of the Study

The NAO robot is one of the most widely used agents for human interaction, and the understanding of human emotional expression underlies most of the applications it is currently involved in. Therefore, it is crucial for the community that wishes to use the NAO robot to understand its accuracy in recognizing human emotional expressions. However, the embedded NAO FER module’s accuracy was not yet tested against an FER-validated system. In the present study, FaceReader 7, an FER-validated software, was used as ground truth to evaluate NAO FER module’s accuracy for each of the facial expressions it is able to detect (Figure 10a and Table 1). 

Moreover, since social robots are intentionally designed to interact with humans in a spontaneous, interpersonal way (often with specific social–emotional goals), it is of paramount importance to test their interaction capability in everyday situations. Consequently, testing the robotic system’s capability to recognize human facial expressions in real-life conditions is essential. Although different, highly accurate CNN-based FER models have been developed in the literature over the last decade [22], their integration into a robotic platform was not yet proven effective. Of note, the CNN architecture reported by Melinte et al., which was successfully integrated into the NAO robotic platform and demonstrated to perform better than the proposed CNN-based FER model in the same task, was not actually evaluated in human–robot interaction conditions [43]. On the other hand, testing the CNN architecture developed and integrated into the robotic platform with human participants would allow one to assess its performance in real-world scenarios, paving the way for its implementation in robotics applications. The innovations of this study were the integration of a CNN-based FER model into the NAO robotic platform and its appraisal in real-world scenarios, thus providing specific metrics related to its implementation in human–robot interaction tasks. Such metrics include the CNN accuracy achieved during robot interaction activities (Figure 10), the accuracy reached with respect to participants’ different facial orientations (Table 2), and the time required to process the facial expression detection and provide this information to the robot’s SDK (0.34 ± 0.04 s). Furthermore, the CNN presented in this study enables, for the first time, one to endow the NAO robot with the capability of the real-time estimation of an interlocutor’s arousal level through a linear combination of CNN outcomes.

Though the proposed CNN-based FER model achieved excellent results and has proven its feasibility in real-world robotic interactions tasks, some improvements may be required to obtain more accurate overall results. Firstly, a higher recognition rate for the angry and scared facial expressions should be achieved. Secondly the CNN’s architecture could be enhanced by employing, for instance, ResNet or Visual Geometry Group (VGG) model-based architectures, which have recently been shown to have great potential in this field [43]. Future research will focus on optimizing the CNN model in order to increase its performance, as well as testing it for complex human–robot interaction tasks such as those in the medical or neuropsychological fields. 

## 5. Conclusions

In this research, a CNN-based FER model was developed for the recognition of facial expressions in real-life situations. The overall system was integrated into a humanoid robot to enable it to deal with challenging real-life facial emotion recognition tasks. The results demonstrated the CNN-based FER model’s ability to surpass the NAO’s FER module’s performance in recognizing emotions based on facial expressions. Furthermore, the implemented model enabled NAO to detect more emotional information than its original module (i.e., the level of arousal and the scared facial expression). This module represents the first stage of an automated empathic behavior system and could pave the way for human-like robot interactions. The implementation of this module could enhance the effectiveness of companion and assistive robots for clinical and social purposes. 

## Figures and Tables

**Table 1:** Precision, recall, and F1 score achieved by NAO’s FER module on the experimental data for each output class compared to those of the CNN-based FER module. The best performances are highlighted in bold.

	Precision		Recall		F1 Score	
	NAO’s FER module	CNN-based FER model	NAO’s FER module	CNN-based FER model	NAO’s FER module	CNN-based FER model
Angry	0.22	**0.46**	**0.61**	0.58	0.32	**0.52**
Scared	-	**0.72**	-	**0.75**	-	**0.73**
Happy	**0.69**	0.44	0.87	**0.91**	**0.77**	0.6
Neutral	0.93	**0.96**	**0.72**	0.68	**0.81**	0.8
Sad	0.63	**0.8**	0.53	**0.9**	0.57	**0.85**
Surprised	0.25	**1**	0.55	**0.75**	0.34	**0.86**

**Table 2:** Percentage of frames where the face was correctly detected and the accuracy achieved with respect to different facial orientation angles.

	Angles (°C)	Accuracy Achieved	Frame Correctly Tracked
Yaw	0 ± 5°	0.83 ± 0.08	99.8% ± 0.2%
	±26 ± 5°	0.38 ± 0.21	94.8% ± 0.3%
Pitch	0 ± 2°	0.84 ± 0.07	99.7% ± 0.2%
	±7 ± 0.96°	0.77 ± 0.11	95.1% ± 0.4%

## Data Availability

The data presented in this study are available on request from the corresponding author. The data are not publicly available due to privacy issues.
